# Three-Dimensionally Preserved Integument Reveals Hydrodynamic Adaptations in the Extinct Marine Lizard *Ectenosaurus* (Reptilia, Mosasauridae)

**DOI:** 10.1371/journal.pone.0027343

**Published:** 2011-11-16

**Authors:** Johan Lindgren, Michael J. Everhart, Michael W. Caldwell

**Affiliations:** 1 Department of Earth and Ecosystem Sciences, Lund University, Lund, Sweden; 2 Sternberg Museum of Natural History, Fort Hays State University, Hays, Kansas, United States of America; 3 Department of Earth and Atmospheric Sciences, and Department of Biological Sciences, University of Alberta, Edmonton, Alberta, Canada; University of Cape Town, South Africa

## Abstract

The physical properties of water and the environment it presents to its inhabitants provide stringent constraints and selection pressures affecting aquatic adaptation and evolution. Mosasaurs (a group of secondarily aquatic reptiles that occupied a broad array of predatory niches in the Cretaceous marine ecosystems about 98–65 million years ago) have traditionally been considered as anguilliform locomotors capable only of generating short bursts of speed during brief ambush pursuits. Here we report on an exceptionally preserved, long-snouted mosasaur (*Ectenosaurus clidastoides*) from the Santonian (Upper Cretaceous) part of the Smoky Hill Chalk Member of the Niobrara Formation in western Kansas, USA, that contains phosphatized remains of the integument displaying both depth and structure. The small, ovoid neck and/or anterior trunk scales exhibit a longitudinal central keel, and are obliquely arrayed into an alternating pattern where neighboring scales overlap one another. Supportive sculpturing in the form of two parallel, longitudinal ridges on the inner scale surface and a complex system of multiple, superimposed layers of straight, cross-woven helical fiber bundles in the underlying dermis, may have served to minimize surface deformation and frictional drag during locomotion. Additional parallel fiber bundles oriented at acute angles to the long axis of the animal presumably provided stiffness in the lateral plane. These features suggest that the anterior torso of *Ectenosaurus* was held somewhat rigid during swimming, thereby limiting propulsive movements to the posterior body and tail.

## Introduction

Mosasaurs are a group of marine reptiles that occupied a wide array of predatory niches in epicontinental seas and shallow oceans worldwide during the latter half of the Cretaceous [1]. Considered to be allied with snakes and lizards [2], [3], mosasaurs have traditionally been portrayed as serpentine animals with slender bodies and elongate, laterally flattened tails [4]–[6]. However, in recent years this view has been challenged in favor of hypotheses suggesting a convergence in body form and caudal fluke morphology with fast, sustained swimmers, such as ichthyosaurs and cetaceans [7]–[11]. Crucial to our understanding of mosasaur evolution, and the degree of aquatic adaptations they achieved, are the preservation of soft-tissue structures, which for a long time were limited to small patches of scales in a few forms [4], [9], [12]–[15]. However, the recognition of fossilized soft-tissues in an exceptionally preserved specimen of *Platecarpus* (LACM 128319; Natural History Museum of Los Angeles County) from the Niobrara Formation of Kansas, USA, provided insights into anatomical features (e.g., anteriorly migrated viscera and downturned tail) that presumably facilitated streamlining and high efficiency swimming [10]. Here we report on another extraordinarily preserved specimen (FHSM VP-401; Sternberg Museum of Natural History) that, for the first time in a mosasaur, allows characterization of not only the epidermal scales but also the underlying dermis and its fiber bundle morphology. Hence, FHSM VP-401 provides information on the deeper structure and mechanical properties of the mosasaur integument. These new data are central to expand our understanding of the degree of aquatic specializations convergently achieved by multiple organ systems in distantly related mosasaurs, in what we now know was a short period of geological time (i.e., less than 10 million years [10]).

## Results

### Systematic Paleontology

Squamata Oppel 1811

Mosasauroidea Camp 1923

Mosasauridae Gervais 1852

Russellosaurina Polcyn & Bell 2005

*Ectenosaurus* Russell 1967

*Ectenosaurus clidastoides* (Merriam 1894)

### Description of Integumentary Structures

**Figure 1:**
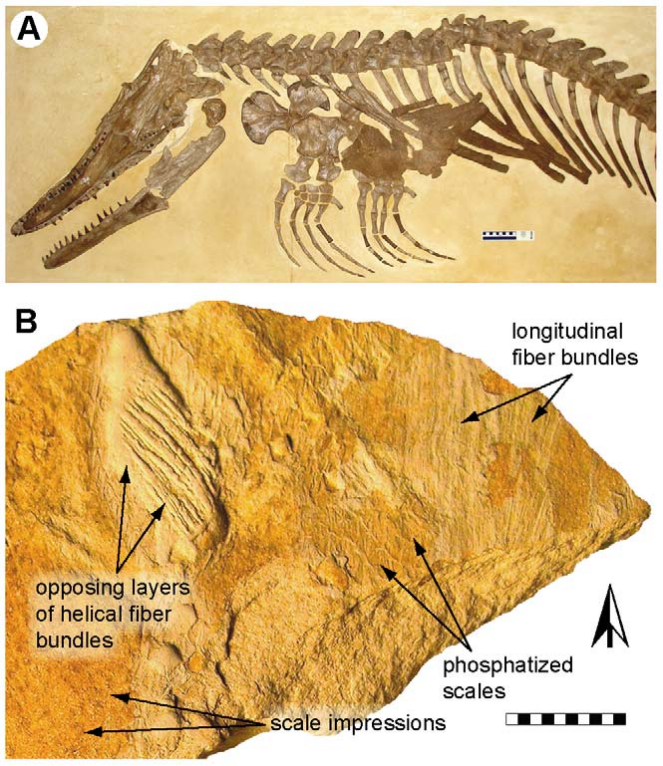
*Ectenosaurus clidastoides* FHSM VP-401. (A) Skull, partial axial and appendicular skeleton, and calcified sternal cartilage in oblique ventro-lateral view. (B) Slab FHSM VP-401-05 showing phosphatized integumentary structures in medial view. Black and white arrow indicates anterior. Scale bars, (A) 10 cm and (B) 10 mm.

**Figure 2:**
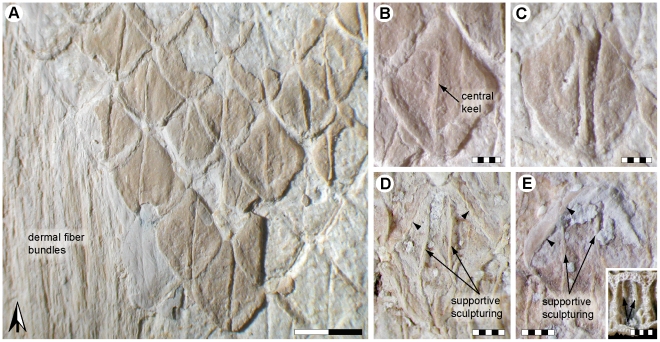
*Ectenosaurus clidastoides* FHSM VP-401. Squamation. (A) External view of imbricating and obliquely arrayed scales (FHSM VP-401-01). Note underlying phosphatized dermal fiber bundles oriented parallel to the long axis of the animal. Black and white arrow indicates anterior. (B) External view of a flattened, rhomboidal scale (FHSM VP-401-01). Note presence of a distinct central keel. (C) External view of a three-dimensional, ovoid scale (FHSM VP-401-01). The parallel furrows adjacent to the central keel probably represent artifacts of preservation. (D) Medial surface of a rhomboid scale (FHSM VP-401-08) showing the interior ridged support of the *ß*-layer of the epidermis (arrows). Note medially inclined scale hinge (arrowheads). (E) Medial surface of an ovoid scale (FHSM VP-401-08) showing the supportive sculpturing of the *ß*-layer of the epidermis (arrows). Note folded scale hinge (arrowheads). Inset, inside of a *Varanus gouldi* body scale (MZLU L867/3039) showing supportive sculpturing (arrows) similar to that seen in *Ectenosaurus*. Scale bars, (A) 2 mm and (B–E) 0.5 mm.

**Figure 3:**
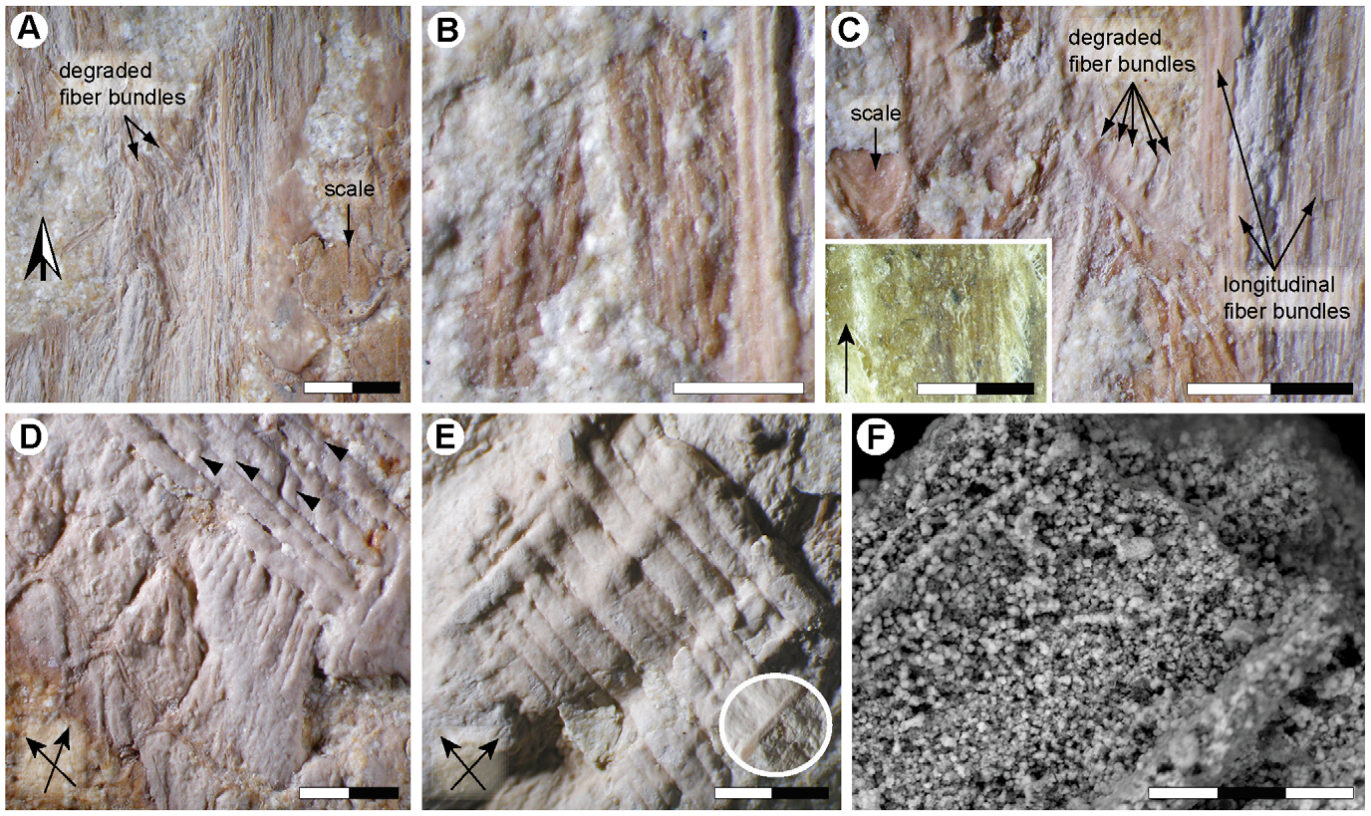
*Ectenosaurus clidastoides* FHSM VP-401. Dermal fiber bundle architecture. (A) FHSM VP-401-01 showing partially degraded, longitudinal fiber bundles underlying epidermal scales. Black and white arrow indicates anterior. (B) Close-up of FHSM VP-401-04 showing superficial layers of fiber bundles with a predominantly longitudinal orientation. Note that the fiber bundles located somewhat deeper in the dermis (at the right hand side of the picture) appear to be somewhat thicker than are those immediately below the epidermal scales (at the center and left hand side of the image). (C) Longitudinally oriented and partly degraded fiber bundles located immediately below the epidermis (FHSM VP-401-04). Inset, longitudinal (arrow) dermal fiber bundles from the neck region of *Eunectes* sp. (LO 11215). (D) Transverse and tangential sections through helically arranged fiber bundles (FHSM VP-401-05). Arrowheads show fiber bundles that appear to be cross-weaved with those of adjacent layers, whereas arrows indicate the principal fiber bundle directions. (E) Layers with fiber bundles that extend in opposing directions (arrows) from deep within the dermis in tangential view (FHSM VP-401-04). Despite being heavily encrusted with phosphate, the fiber bundles are still readily visible. Inset (also in tangential view), a better preserved fiber bundle from the base of the section. (F) Backscatter image of an isolated fiber bundle comprised of multiple apatite aggregates (FHSM VP-401-01). Scale bars, (A, C–E) 2 mm, (B) 1 mm and (F) 30 µm.

FHSM VP-401 was collected in 1953 from the Santonian (Upper Cretaceous) part of the Smoky Hill Chalk Member of the Niobrara Formation ‘on Garrett Ranch, seven or eight miles northwest of Wakeeney, Trego County, Kansas,’ USA, by George F. Sternberg ([1] p. 158). The specimen is comprised of a complete skull and articulated anterior half of a skeleton (Figure 1A) belonging to an unusual, long-snouted russellosaurine mosasaur, denominated *Ectenosaurus* (from *Ectenes*, Gr., drawn-out; *sauros*, Gr., lizard) by Russell [1]. Associated with the skeletal and cartilaginous remains are 23 small slabs preserving fossilized integument (Figures 1B, 2, 3). Although Russell ([1] p. 70) reported ‘rhomboid scales’ and Everhart ([16] p. 167) mentioned ‘skin impressions’, these soft-tissue structures have hitherto remained unstudied. Because the skeletal anatomy of *Ectenosaurus* is reasonably well known (see [1] for description), the exceptionally preserved dermal covering of FHSM VP-401 is the focus of this report.

A total of 23 chalk slabs (FHSM VP-401-01–FHSM VP-401-23) ranging in size from about 25×40 to 105×125 mm display portions of integument. The squamation is preserved as articulated sections of phosphatized matter and as a dark, reticulated pigmentation on the calcareous matrix that surrounded FHSM VP-401 (Figures 1B, 2 and 3A, C, D). The scales are roughly uniform in size (measuring about 2.7 mm in length and 2.0 mm in width) and obliquely arrayed into an alternating pattern where neighboring scales overlap one another (Figure 2A). Most scales are flattened, with a symmetrical, distinctly rhomboidal outline (Figure 2B); however, the latter is presumably an artifact of preservation because all scales that retain relief have a more or less ovoid shape (Figure 2C). From the three-dimensionally preserved scales it is further established that each scale was originally gently vaulted. A central, longitudinal keel divides the external surface of each scale into two halves of equal size (Figure 2A–C). The inner scale surface exhibits two parallel and longitudinal ridges that presumably represent supportive sculpturing of the *ß*-layer (i.e., outer layer) of the epidermis (Figure 2D, E—arrows). In medial view, each scale is surrounded by a thin, smoothly-surfaced wall, i.e., the scale hinge, which is either acutely bent medially (Figure 2D—arrowheads) or folded inwards (Figure 2E—arrowheads); the latter condition is presumably a result of compression from the weight of overlying sediments.

Subjacent to the scales are layers of pale, strand-like structures exposed in both transverse (i.e., at right angles to the skin surface) and tangential (i.e., parallel to the skin surface) views (Figures 1B, 2A and 3). Some of the strands are flattened (Figure 3A); others retain much of their presumed original three-dimensional form (Figure 3B). By reference to extant vertebrates [17]–[19], it is here assumed that the strands represent the fossilized remains of structural fiber bundles from the dermis. The thickest fiber bundles are located deepest in the skin (i.e., approximately 2 mm below the skin surface in its present, somewhat compressed state) and the thinnest are the outermost; however, this may be a taphonomic artifact because the outer layers generally are better preserved than are the deeper ones (cf. Figure 3B, E). Well-preserved fiber bundles measure about 90 µm in diameter, and occasionally show signs of decay, such as folding (due to loss of tension) and branching patterns (Figure 3A, C). The fiber bundles are either straight, densely spaced and oriented at acute angles (i.e., almost parallel) to the long axis (determined from the shape and arrangement of the scales) of the animal (Figures 2A, 3A–C), or are arranged in tightly stacked layers with alternating left- and right-handed orientations (a crossed-helical architecture; Figure 3D, E). A minimum of eight layers with helically arranged fiber bundles are preserved, and the fiber angles are in the range of 20–70° to the long axis of the animal (the predominant fiber angles are in the 40–55° range). Additionally, some fiber bundles appear to cross-weave with those of adjacent layers (Figure 3D—arrowheads).

Under SEM it was observed that the fiber bundles are comprised of micrometer-sized bodies with morphologies similar to those of apatite crystallites (Figure 3F; cf. [20]). This finding was corroborated with EDX point sum spectrum analysis, which revealed distinct peaks in C, Ca and P, to suggest a primary composition of calcium phosphate.

## Discussion

The vertebrate integument serves a number of important biological and mechanical roles, including e.g., protection against predation and parasites, support of the enclosed body contents and control of water loss [21]–[23]. Additionally, it provides a strong yet flexible covering that allows changes in body shape occurring during locomotion, or as a means of resisting changes to body shape resulting from muscular activity and movement [18], [19], [21]. These dynamic functions are largely determined by structural characteristics of the dermis, which in turn are dependent on a complex meshwork of collagen and elastin fibers [24]. The mechanical properties of skin and associated fibrous tissues have been examined in an array of extant vertebrate taxa, including bony fish [21], sharks [17]–[19], reptiles [17], cetaceans [25], [26], and birds [17]; however, given sparse preservation of soft-tissue structures (other than scales, hairs and feathers) our knowledge of the integumental fiber architecture in fossil vertebrates is hitherto limited to ichthyosaurs (e.g., [17], [27], [28]), pterosaurs (e.g., [29]) and dinosaurs (e.g., [22]). Hence, the discovery of an elaborate system of multiple-layered fiber bundles in FHSM VP-401 constitutes a significant development in so far as it represents the first unambiguous record of deeper soft-tissue structures in the skin of an extinct squamate (but see also [9]).

The epidermal scales of FHSM VP-401 show relief and, for the first time in a mosasaur, can be examined in both lateral and medial view. Although the precise locations of the scales (and subjacent integumental structures) are unknown, their uniform shape and diminutive proportions suggest that they originate from the neck and/or trunk of the animal (cf. [10]); this is also in accordance with an unsigned note in pencil, possibly George F. Sternberg's handwriting, indicating that the scale slabs came from the neck region of FHSM VP-401. Whereas hydrodynamic aspects of keeled body scales have been dealt with elsewhere ([9] and references therein), the supportive sculpturing on the underside of the scales has previously not been reported in mosasaurs [although the proposed ‘multiple keels’ on the body scales of *Plotosaurus* (see [9]) may in fact be supportive sculpturing]. Supportive structures are present on the inside of osteoderms and scales in certain nonavian dinosaurs [30] and in many extant lizards [31]; notably, two parallel, longitudinal crests occur on larger body scales of some monitor lizards (Figure 2E, inset). Assuming that the longitudinal ridges act as attachment sites for underlying ligaments or connective tissue, they may serve a function as anchors, thereby providing strength to the skin [30].

The scales of FHSM VP-401 are considerably smaller in size (2.7×2.0 mm) than are those of LACM 128319 (*Platecarpus*; average scale size is 3.8×4.4 mm), despite a comparable estimated total body length of the two animals (5.9 m versus 5.7 m [10]). Additionally, in a specimen of *Tylosaurus* (KUVP 1075; Natural History Museum and Biodiversity Research Center) the scales are 3.3×2.5 mm, hence placing it firmly between FHSM VP-401 and LACM 128319. Snow ([12] p. 57) described the tylosaur as a ‘small-sized individual of its species’ but gave no measurements to substantiate that description other than the length of the humerus (12.2 cm). Based on a comparison with a 9 m specimen of *Tylosaurus proriger* (FHSM VP-3) where the humerus is approximately 22 cm in length, we estimate that KUVP 1075 originally measured about 5 m in overall body length. Given that extant lizards generally hatch with a fixed number of scales which then grow in size with each molt (resulting in larger scales in older individuals [32]), the discrepancy in scale size that we have recorded may be the result of age differences between the three individuals represented by FHSM VP-401, KUVP 1075 and LACM 128319. However, such a scenario would imply that FHSM VP-401 represents a younger individual than does LACM 128319, and that *Ectenosaurus* could reach body lengths well beyond those of *Platecarpus*, something that has yet to be proven (a few large-sized but otherwise enigmatic specimens from the Smoky Hill Chalk, such as KUVP 1024, may in fact belong to *Ectenosaurus*). Another possibility is that the scales come from different parts of the body on, at least, FHSM VP-401 and KUVP 1075. It is also possible that the two plioplatecarpine genera discussed here (i.e., *Ectenosaurus* and *Platecarpus*), considering their distant relationship to each other [33], [34], may have had different-sized scales to begin with.

Given its narrow, elongate skull and slender teeth, we may assume that *Ectenosaurus* was primarily piscivorous, and thus benefited from possessing a squamation comprised of small-sized, firmly anchored and keeled body scales that presumably contributed to an anterior-posterior channeling of the water flow (cf. [9]), thereby reducing frictional drag when trying to overtake smaller, more streamlined prey. Surface deformation (and thereby frictional drag), may have been further reduced by the cross-woven helical fiber bundles in the subjacent dermis. A similar fiber arrangement has been observed in some extant sharks [35] and the Burmese python [17], in regions of the body that are likely to face considerable stress. In large aquatic vertebrates, such as ichthyosaurs [27], [28], [36], [37], sharks [18], [19], [35] and dolphins [25], [26], [38], straight (i.e., high tensile) fiber bundles are often organized into multiple-layered helical networks. Presumably, this arrangement minimizes creasing of the skin, thereby counteracting fluid drag by retaining a smooth body surface. Assuming that the majority of the measured fiber angles are primary (rather than the result of taphonomic changes from e.g., microbial and/or chemical degradation), then those representing the crossed-helical fiber bundles are, on average, somewhat smaller than are fiber angles from the anterior trunk of tunas [21] and ichthyosaurs [27], [37], but equal to those of the subdermal connective tissue sheath of dolphins [25]. Given that the mechanical function of skin varies over different parts of an animal's body, the generally low fiber angles recorded for *Ectenosaurus* could indicate that a large amount of the preserved integument originates from the ventral face of the neck and/or trunk, as this part normally experiences lower strain than does the back (cf. [22]). Again, this conclusion is corroborated by George F. Sternberg's notes, which state that the integument probably derives from the area between the mandible and chest/limb; i.e., on the lower half of the animal.

Of particular interest are those fiber bundles that are oriented sub-parallel to the long axis of FHSM VP-401 (Figures 1B, 2A and 3A–C). Laterally, these layers seem to alternate with layers showing a crossed-helical fiber bundle architecture (Figure 1B), although this may be an artifact of preservation. Parallel-oriented, longitudinal fiber bundles have previously been reported in the skin of macrostomatan snakes, such as *Eunectes* (Figure 3C, inset), and in ichthyosaurs. Whereas the skin of macrostomatan snakes shows features facilitating the consumption of large prey items [39], longitudinally arranged fiber bundles presumably provided stiffness in the lateral plane and counteracted torsional stresses in the integument of ichthyosaurs [28], [37]. A similar reinforcing function is likely the case in *Ectenosaurus*, given that both mosasaurs and ichthyosaurs inhabited the marine realm and thus faced comparable hydrodynamic constraints imposed by the surrounding water.

The combination of small-sized, firmly anchored body scales and a complex meshwork of alternating crossed-helical and longitudinal fiber bundles suggests a stiffening of the anterior torso in *Ectenosaurus*. Thus, it is reasonable to assume that this part of the body was held somewhat rigid during locomotion, whereas the lateral thrust-producing flexure was restricted to the posterior trunk and tail. Accordingly, *Ectenosaurus* probably utilized a sub-carangiform rather than an anguilliform mode of swimming (see e.g., [40] for categories of swimming styles).

## Materials and Methods

The fossilized integument of FHSM VP-401 and skin samples from a number of extant squamates, including e.g., *Varanus gouldi* (MZLU L867/3039; Museum of Zoology, Lund University; Figure 2E, inset) and *Eunectes* sp. (LO 11215; Department of Earth and Ecosystem Sciences, Lund University; Figure 3C, inset), were examined and photodocumented using a Nikon Coolpix 990 camera attached to a Nikon SMZ1000 binocular microscope. Dermal fiber bundles illustrated in Figure 3A–C were brushed with water to increase contrast prior to being photographed. Samples selected for scanning electron microscopic (SEM) analysis were mounted on glass slides using double-sided carbon tape and examined uncoated under low vacuum using a Hitachi S-3400N scanning electron microscope. Energy dispersive X-ray analysis (EDX) was used to establish the elemental composition of both isolated scales and fiber bundles. The terminology is based on that of [17], [31], and the systematics follow that of [33].
